# How Cell Geometry and Cellular Patterning Influence Tissue Stiffness

**DOI:** 10.3390/ijms23105651

**Published:** 2022-05-18

**Authors:** Mateusz Majda, Nicola Trozzi, Gabriella Mosca, Richard S. Smith

**Affiliations:** 1 Department of Computational and Systems Biology, John Innes Centre, Norwich NR4 7UH, UK; nicola.trozzi@jic.ac.uk; 2 Department of Comparative Development and Genetics, Max Planck Institute for Plant Breeding Research, 50829 Cologne, Germany; mosca@mpipz.mpg.de; 3 Department of Physics, Technical University Munich, 85748 Garching b. München, Germany

**Keywords:** cell geometry, cellular patterning, cell growth, biomechanics, microextensometer, finite element method, FEM, modeling, MorphoRobotX, MorphoMechanX

## Abstract

Cell growth in plants occurs due to relaxation of the cell wall in response to mechanical forces generated by turgor pressure. Growth can be anisotropic, with the principal direction of growth often correlating with the direction of lower stiffness of the cell wall. However, extensometer experiments on onion epidermal peels have shown that the tissue is stiffer in the principal direction of growth. Here, we used a combination of microextensometer experiments on epidermal onion peels and finite element method (FEM) modeling to investigate how cell geometry and cellular patterning affects mechanical measurements made at the tissue level. Simulations with isotropic cell-wall material parameters showed that the orientation of elongated cells influences tissue apparent stiffness, with the tissue appearing much softer in the transverse versus the longitudinal directions. Our simulations suggest that although extensometer experiments show that the onion tissue is stiffer when stretched in the longitudinal direction, the effect of cellular geometry means that the wall is in fact softer in this direction, matching the primary growth direction of the cells.

## 1. Introduction

Plant growth is driven by the turgor pressure within cells that generates tensile stress on the surrounding walls. Cell walls are located outside the plasma membrane and consist of load-bearing cellulose microfibrils embedded in matrix polysaccharides, such as highly hydrated pectins, hemicelluloses and structural proteins [1]. They must be stiff enough to resist the high turgor pressure within the cell, yet must also be able to expand dramatically in growing cells. Thus, cell walls are dynamic and need to modify their properties during plant development [2]. The stretch induced by turgor is elastic, and when combined with cell-wall modifiers that make this elastic strain plastic, the cells grow [3]. As reflected in the Lockhart model of plant cell growth, the amount of growth is a combination of how much the cell wall is stretched by turgor (its elasticity) and the presence of growth factors that modify the cell wall and make it extensible [4,5,6]. Although it is widely believed that growth is correlated with cell-wall stiffness, this is not always the case. The xyloglucan-deficient cell walls in *xxt1xxt2* hypocotyls are softer and weaker yet are less extensible [3,7]. Nevertheless, the relationship between growth and stiffness provides a straightforward explanation for the emergence of elongated cell shapes via anisotropic cell-wall properties. When the cell wall is reinforced by cellulose in a specific direction, turgor causes more stretch in the softer direction. In this scenario, the expression of cell-wall-loosening growth factors causes the cell to elongate preferentially in the softer direction, perpendicular to the principal cellulose orientation. Alternatively, it is possible that cell-wall looseners act to allow the cell wall to extend in the same direction as the orientation of the cellulose, by allowing the fibers to slide with respect to each other [3]. Both models rely on the orientation of cellulose microfibrils to provide the directional information for anisotropic growth, with a stiffness-based model growing more perpendicular to the primary orientation, and a sliding model growing more parallel to it. Interestingly, this shows a curious parallel to the abstract notions of parallel and perpendicular growth rates used in simulation models of leaf form [8], where growth factors are thought to act either parallel or perpendicular to tissue polarity induced by gradients of morphogens.

Since growth depends on cell stress and the strain it causes, cellular geometry and arrangement can have a significant impact on growth. In the *Arabidopsis* embryo, tightly packed cells at the tip grow less, even though they are more abundant in growth-promoting genes than more distal cells [9]. This happens because smaller cells have less elastic strain than larger cells with the same turgor pressure, so it is expected that larger cells would grow more given the same amount of cell wall extensibility factors. Since plant cells are attached to each other by their rigid cell walls, growth is also affected by neighboring cells [10]. This implies that the arrangement of cells within tissues can affect the development of organ shape and form. Shape can also have an anisotropic effect on stress, as elongated cells have more stress in the transverse direction than the longitudinal, and have more elastic strain due to turgor if the cell wall is isotropic. Thus, cell growth is not only affected by turgor, wall elasticity and the presence of extensibility factors, but also by cell size and shape and its context within a tissue [11,12,13].

Several different methods have been used to measure mechanical properties at the subcellular, cellular and tissue levels; however, mechanical measurements need to be carefully interpreted as they can be influenced by external forces, cell turgor pressure and geometry and patterning at the cellular, tissue and organ levels [14]. To separate the effects of these different factors, finite element method (FEM) models of the cellular tissue can be used to simulate the experiments e.g., [11,15,16,17].

Microindentation has been used to measure cell-wall stiffness and turgor pressure by applying a force with a mechanical probe at given displacement and recording the force–indentation curves. The most widely used indentation method is atomic force microscopy (AFM), which measures subcellular stiffness (elasticity) [18,19,20,21]. AFMs were originally equipped with probes having nanoscopic tips to measure very low mechanical forces at the molecular and atomic levels. Bigger and stiffer probes were later introduced to study plant tissues [22,23]. Progressing from nano- to microindentation, cellular force microscopy (CFM) is designed to measure forces in plants at the cellular scale, and is equipped with bigger probes, measuring much higher forces and being able to indent with larger displacements on much larger cell regions [24,25]. A recently emerging method is the nondestructive contact-free Brillouin microscopy, which analyzes the optical frequency shift of scattered light to estimate material properties [26,27]. Note that all these methods make measurements in the direction normal to the surface and are not able to determine cell-wall in-plane stiffness and anisotropy, which are the parameters most relevant for growth.

To directly measure tissue stiffness, an extensometer can be used to stretch the tissue while simultaneously measuring the force. By applying forces over a period of time and measuring the creep, cell-wall extensibility can also be measured. The sample is clamped and stretched with the extensometer, and force–displacement curves are recorded and used to determine the material stiffness. The method has broad applications in life sciences, having been used on both plant and animal samples, and has recently been used with confocal microscopy to study forces at the cellular resolution [28,29,30,31,32]. It has been used to stretch *Drosophila* imaginal wing disks, where it was shown that tensional stress increases cell proliferation rates [33], and to study the microtubule response to stress in *Arabidopsis* hypocotyl cells [32]. Direct mechanical measurements can also be obtained using a pressure probe. This approach is used to manipulate turgor pressure and measure the resulting deformation at the single-cell level [34]. Another popular method to measure cell and tissue stiffness is osmotic treatment. Osmotic treatments are used to manipulate turgor pressure and induce tissue deformation, which is then tracked with confocal time-lapse microscopy, providing estimates of cell-wall stiffness [16,35]. The extensometer, pressure probe and osmotic treatment methods can all measure stiffness in the in-plane directions (i.e., not just normal to the surface), which are the directions most relevant for growth.

Epidermal onion peels have been used as a convenient model system to study plant cell-wall stiffness and cell-wall material anisotropy, as they are easily detachable from the whole organ and they provide an intact single layer of staggered cells [14,36,37,38,39,40,41]. Results from extensometer experiments are often interpreted from the perspective of the tissue as a continuous solid and show that the longitudinal direction is stiffer than the transverse one [39,42]. This is contrary to what would be expected for cell-wall anisotropy of elongated cells, where one would expect the tissue to be softer in the primary growth direction. Here, we hypothesized that the tissue-level measurements may not be directly reflecting the stiffness of the cell wall, and that the geometry and arrangement of the cells may affect the tissue-level apparent stiffness in stretching experiments. To test this hypothesis, we used a microextensometer to stretch onion epidermal sections in the longitudinal and transverse directions, and in solutions of different osmolarity. We then performed FEM simulations of the extensometer experiments on 3D pressurized idealizations of the onion epidermis, to understand the contribution from cellular geometry and arrangement on the forces measured in vivo. This enabled us to estimate how the stiffness and anisotropy of the cell-wall maps to the whole-tissue measurements obtained from extensometer experiments.

## 2. Results and Discussion

### 2.1. Tissue Mechanical Stiffness Is Anisotropic

**Figure 1:**
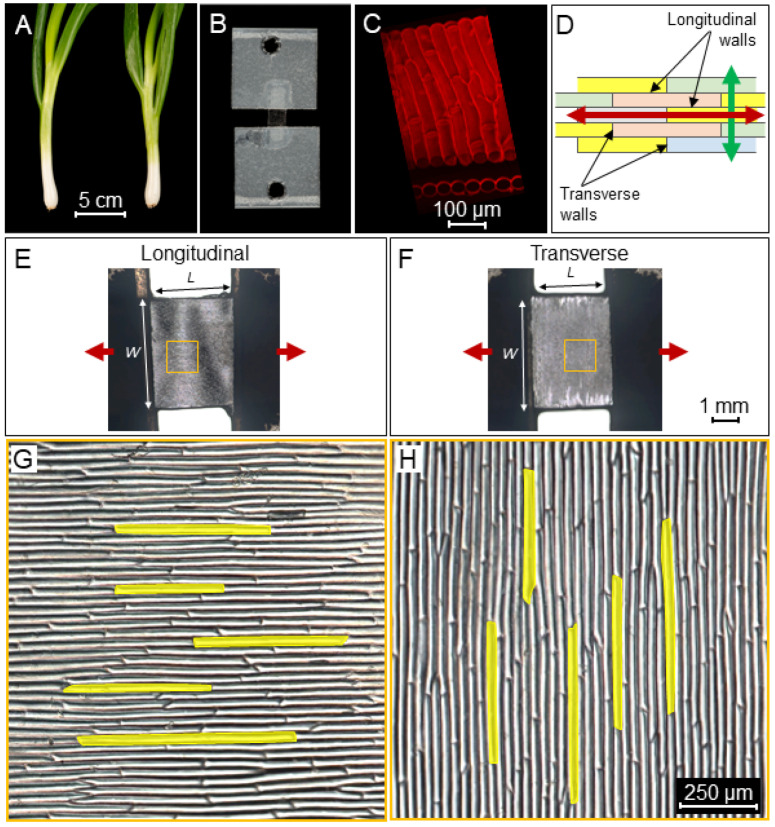
Preparation of longitudinal and transverse sections for tensile testing with a microextensometer. (**A**) The adaxial onion epidermal peel was obtained from white bulbs of spring onions. (**B**) Experimental setup of onion epidermal samples attached to Tough-Tags™ for tensile testing. (**C**) The morphology of onion epidermal cells stained with propidium iodide: surface view (top), cross section (bottom). (**D**) The anisotropic cells displaying longitudinal and transverse cell walls were stretched in the longitudinal (red arrow) and transverse (green arrow) directions. (**E**–**H**) Images showing samples for tensile testing arranged in the longitudinal (**E**,**G**) and transverse (**F**,**H**) directions. Red arrows indicate the direction of stretching. (**E**,**F**) Samples were carefully sectioned and measured before the experiment to retain similar size between the two stretching directions. (**G**,**H**) Close-up of the samples from the yellow squares (in **E**,**F**), with visible single-cell outlines indicate that the two samples (**E**,**G**) and (**F**,**H**) consist of cells, which are similar in size. Examples of cell outlines are highlighted in yellow. Scale bars are displayed in the figure.

**Figure 2:**
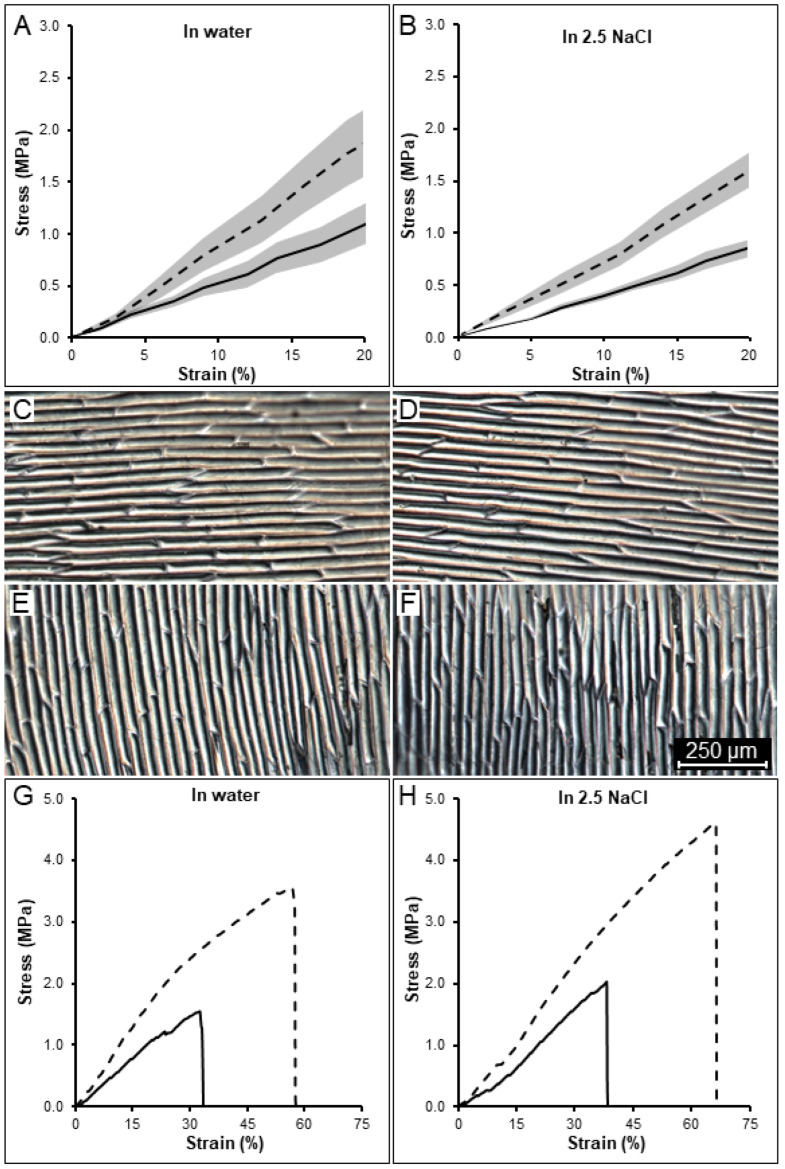
Tensile testing of onion epidermal tissues oriented in longitudinal and transverse directions. Tissue stretching in turgid (**A**,**C**,**E**,**G**) and plasmolyzed (**B**,**D**,**F**,**H**) conditions. (**A**,**B**,**G**,**H**) Normalized strain–stress curves generated from the microextensometer experiment in the longitudinal (dashed line) and transverse (solid line) directions for all samples (**A**,**B**) and a typical single sample (**G**,**H**). The grey ranges indicate the confidence intervals based on standard errors (**C**–**F**) Close-up of the adaxial onion epidermal peels used for tensile testing showing the cell size and patterning. The scale bar is shown in the figure.

To understand how cell geometry affects tissue stiffness, epidermal peels were dissected from young spring onions. The samples consisted of long, thin cells with an average aspect ratio of 20-1 (Figure 1) which were then stretched with a microextensometer (Figure S1) in the longitudinal (Figure 1E,G) and transverse (Figure 1F,H) directions. To determine the stiffness, a force sensor was attached at one end of the sample with the other end of the sample attached to a fixed base, and force–displacement curves were acquired (Figure 2 and Figures S2 and S3). The raw curve of a typical measurement is characterized by a flat region with displacement at a very low force close to the base line (not shown). This region represents the displacement needed to straighten the sample and does not reflect sample stiffness. Once the sample aligns and starts to stretch, the force increases (Figures S2 and S3). At this stage, the stretch applied on the sample is mostly elastic. As the displacement continues, the force increases, until at some point, the strain becomes plastic, and the sample is no longer able to return to its original length (irreversible deformation). In the final stage, the force curve flattens just before the sample breaks. To compare force curves from different sections, tissue stress was calculated from the force normalized over the sample cross-sectional area (width and thickness), while the displacement was calculated as strain in percentage relative to the original sample length.

The longitudinal samples reached a maximum average stress prior rupture of 5.09 MPa (*n* = 5, SE ± 0.83) at 66.3% strain from their original size. The transverse samples reached an average stress prior rupture of 2.46 MPa (*n* = 5, SE ± 0.44) at 48.7% strain. To be able to compare the stress levels between samples and directions, a strain of 20% was chosen as a reference point, which was purely elastic and below the rupture point for all the samples. At this strain, the stresses of longitudinal samples were at an average of 1.78 MPa (Figure 2A, dashed line) and those of transverse ones were at an average of 1.05 MPa (Figure 2A, solid line). While the initial cell size was similar between longitudinal (Figure 2C) and transverse sections (Figure 2E), tissue stress was 1.69 times higher in the longitudinal samples at 20% strain. The higher stiffness observed in these samples is consistent with previous tensile-testing experiments performed on onion epidermis [39,42]. Moreover, these sections break at higher strain than the transverse ones, indicating that cells oriented in the longitudinal direction can deform on average 17.6% more before breaking than cells in the transverse direction (Figure 2G and Figure S2). Thus, it appears that the longitudinal direction is stiffer, yet more deformable.

To understand the role turgor pressure plays in determining the differences in stiffness between longitudinal and transverse stretches, we performed osmotic treatments by placing the onion sections in 2.5 M NaCl for half an hour prior stretching. Placing the sample in the osmoticum removes the influence of turgor pressure on tissue stiffness. The osmotically treated samples (Figure 2B) showed on average a stress value prior to rupture of 5.16 MPa (*n* = 5, SE ± 0.45) and 2.19 MPa (*n* = 5, SE ± 0.20) with a strain of 74.7% and 51%, respectively, for the longitudinal and transverse samples (not shown). At the reference point of 20% strain, samples reached an average stress of 1.65 MPa and 0.84 MPa, respectively, with the longitudinal ones being 1.97 times more stressed. Interestingly, the plasmolyzed samples were able to further stretch on average 8.3% in the longitudinal and 2.3% in the transverse direction before rupturing, and displayed a 1.17 times higher ratio at 20% strain compared to the turgid samples (Figure 2A), consistently among different samples (Figure 2H and Figure S3). Thus, plasmolyzed cells can withstand increased stretching before rupture occurs. Additionally, the use of selective cell-wall digestive enzymes could address the question on how different cell-wall components influence the cell-wall mechanical strength.

### 2.2. A Simulation Model of the Extensometer Experiments

To better understand the difference seen in microextensometer experiments, FEM modeling was performed on idealized cell templates. We began with templates having a 5-1 aspect ratio for comparison with previous work [39,42]. Initially starting from an array of boxes, we rounded the cell sides and ends, with the cells connected in a band with a width approximately 1/3 of the depth of cells. Cells were given isotropic material properties with a Young’s modulus of 100 MPa. The template was then pressurized to 0.5 MPa, causing the cells to expand approximately 15% in volume.

**Figure 3:**
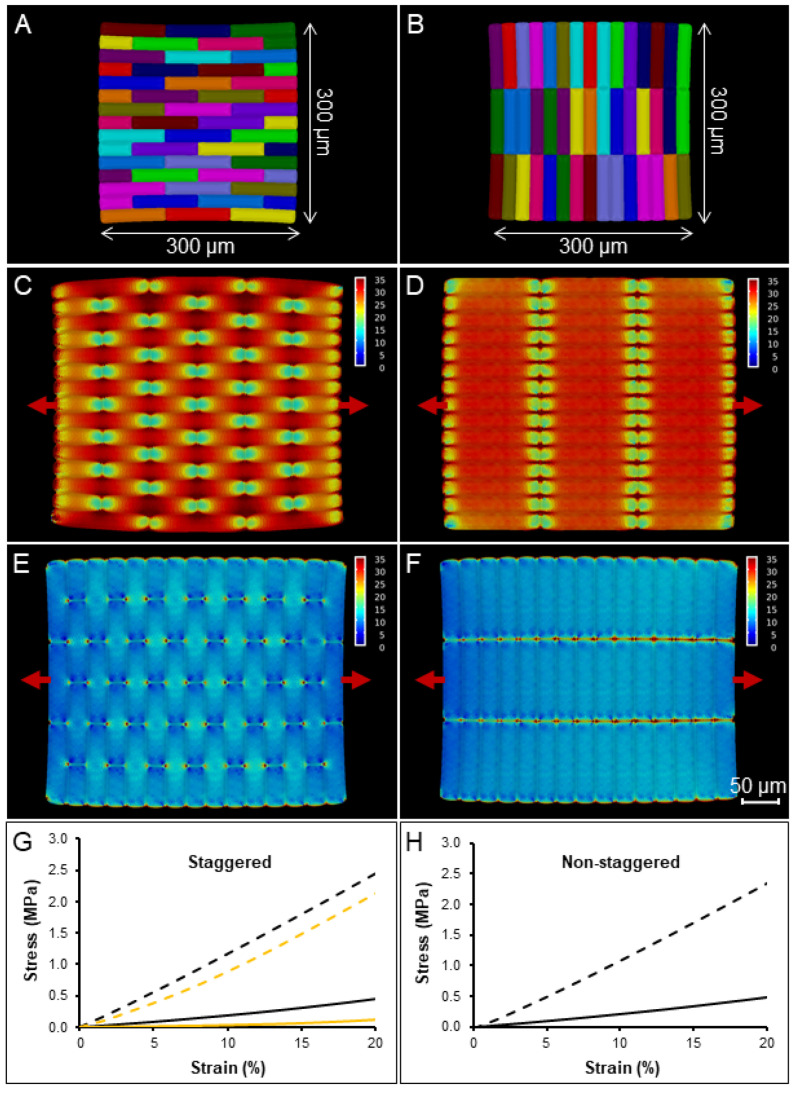
FEM simulation of a tissue with and without cell staggering and anisotropic material properties. (**A**,**B**) The cell templates were generated with a 5-1 cell ratio in CellMaker. The template used for the simulation was cut to a standard size of 300 × 300 µm in a staggered (**A**,**C**,**E**,**G**) or non-staggered (**B**,**D**,**F**,**H**) patterns. (**C**,**D**) The longitudinal and (**E**,**F**) transverse stretch. The heatmaps show the intracellular mechanical stress (trace of Cauchy stress tensor) at 20% strain in the scale from 0 (in blue) to 35 mN (in red). Red arrows indicate the direction of stretch. (**G**,**H**) Stress–strain curves generated from the FEM simulation in MorphoMechanX for longitudinal (dashed lines) and transverse (solid lines) stretch. Simulation of the staggered tissue stretching was performed also in plasmolyzed conditions (**G**, yellow lines). The scale bar is shown in the figure.

We arranged the cells in our initial tissue template in staggered (Figure 3A) and nonstaggered (Figure 3B) patterns and stretched them in the longitudinal (Figure 3C,E) and transverse (Figure 3D,F) directions. The stresses at the cellular level were visualized (from 0 MPa in blue to 35 MPa in red) and represented a combination of stresses induced by turgor and stretching, although the extensometer-induced forces quickly become dominant as the sample was stretched. Force–displacement curves were acquired and converted to stress–strain (Figure 3G,H). To also explore the effect of cell arrangement, we compared both staggered (Figure 3A) and non-staggered templates (Figure 3B). In both arrangements, the stress required to deform the tissue longitudinally was higher (Figure 3G,H dashed lines) than for the transverse direction (Figure 3G,H solid lines). At the reference strain of 20% (Figure 3G,H), longitudinally stretched samples reached 2.44 MPa and 2.34 MPa stress, while transverse ones reached 0.45 MPa and 0.49 MPa stress with a ratio of 5.4 and 4.8, respectively, for the staggered and non-staggered patterns. Stretching of plasmolyzed cells was also simulated in the staggered pattern using 0.01 MPa pressure (Figure 3G, yellow lines). The stress observed at 20% strain was 2.13 MPa and 0.12 MPa for the longitudinal and transverse directions, respectively, with a stress ratio of 17.5. The results show that staggering makes little difference to the apparent tissue stiffness in either direction on a template with elongated cells with a 5-1 aspect ratio. Turgor pressure, conversely, appears to predominately affect tissue stiffness in the transverse direction, where it is also expected to generate more stress at the cellular level.

### 2.3. The Impact of Cell Aspect Ratio and Staggering on Stiffness and Nonlinearity

**Figure 4:**
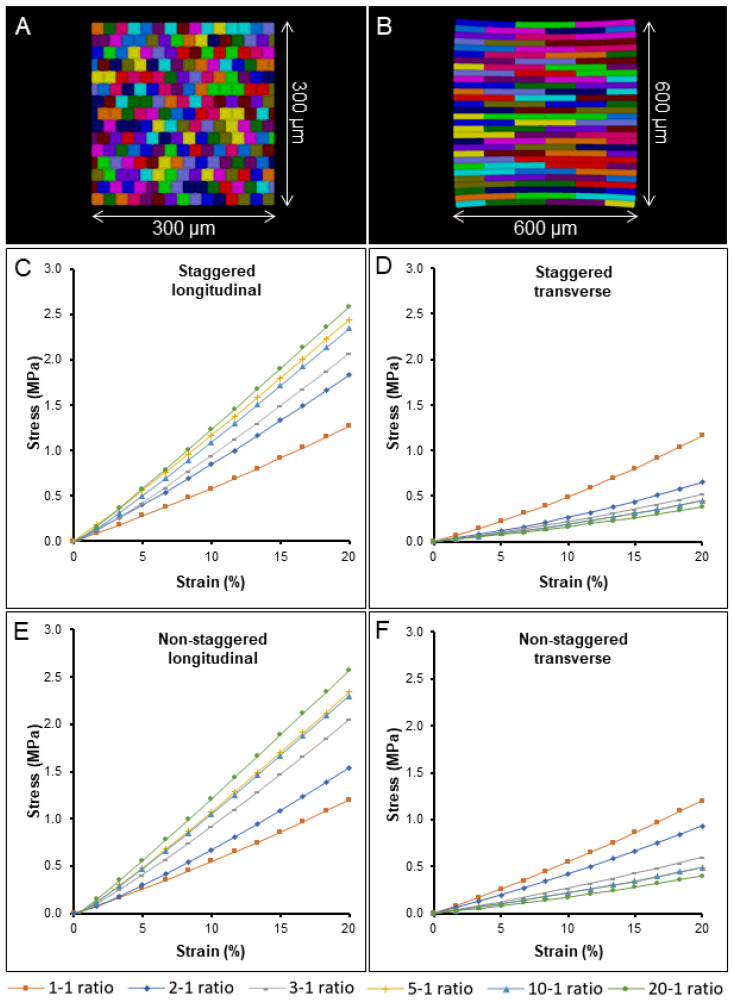
FEM simulation testing how cell–cell arrangement and cell ratio influences tissue stiffness. (**A**,**B**) Cell templates were generated in CellMaker from a cell aspect ratio of 1-1 (**A**) to 20-1 (**B**). The template used for the simulations was cut to a standard size of 300 × 300 um, except for the 20-1 cell aspect ratio of 600 × 600 um. Tissue consisting of cells arranged in staggered (**A**–**D**) or nonstaggered (**E**,**F**) patterns. Tissues stretched in longitudinal (**C**,**E**) and transverse (**D**,**F**) directions. The legend for cell aspect ratios is displayed in the figure.

The impact of cell aspect ratio on tissue stiffness was further tested using templates with different cell aspect ratios ranging from 1-1 for square-shaped cells (Figure 4A) to 20-1 (Figure 4B) for highly elongated cells, which simulates the average aspect ratio of onion cells studied in our experiments (Figure 1G,H). The simulations showed a gradual stiffening for longitudinally stretched tissues and softening for transverse tissues from lower to higher aspect ratios, with the difference becoming progressively smaller for highly elongated cells (Figure 4C,D). The impact cell staggering had on the tissue stiffness ratio (longitudinal over transverse tissue stiffness) also decreased dramatically, from 41.3% with the 2-1 aspect ratio to 5.4% with the 20-1 ratio between staggered and non-staggered samples (Figure 4 compare C,D with E,F). The results indicate that cell staggering of elongated cells has little influence on the overall tissue stiffness and is more important for shorter cells with shapes close to a square. Notably, cell staggering reduces transverse mechanical stress by an average of 14.2%, and our experimental data showed that onion tissues can withstand less stress when stretched transversely (Figure 2G,H solid lines). This supports the idea that cell staggering plays a role in strengthening onion tissues in the transverse direction [14]. The longitudinal stress–strain curves become more linear as the aspect ratio increases, whereas the transverse direction shows the opposite behavior. For the elongated cells used in most experimental work of 5-1 or greater, the longitudinal direction is almost linear, whereas the transverse direction shows substantial strain stiffening. This is likely due to cell flattening as they are stretched, an effect that is more prominent in the transverse direction where there are more cell–cell connections.

### 2.4. Simulation on a Template with Realistic Cell Shapes

**Figure 5:**
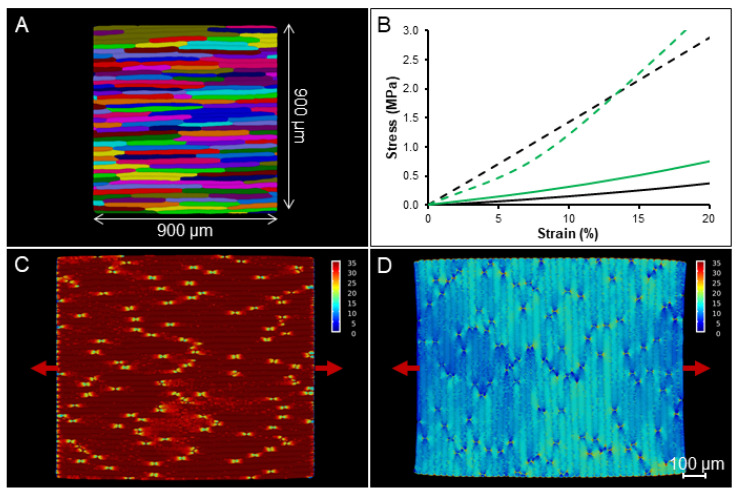
FEM simulation of the realistic onion template. (**A**) Cells segmented from confocal images of adaxial onion epidermis. The template used for the simulation was cut to a standard size of 900 × 900 µm. (**B**) Stress–strain curves generated from the FEM simulation in MorphoMechanX for the longitudinal (**B** dashed line, **C**) and transverse (**B** solid line, **D**) stretch. Black lines represent the simulations run with the engineering strain tensor, while green lines represent the simulations run with the Green–Lagrange strain tensor. (**C**,**D**) The images represent stretched tissue sections at 20% strain. The heatmaps show the mechanical stress (trace of Cauchy stress tensor). Red arrows indicate the direction of stretch. The scale bar is shown in the figure.

To explore if other aspects of cell shape and arrangement not captured by the idealized templates are important for the interpretation of experiments, a tissue template was generated from a confocal image of onion epidermal cells segmented in MorphoGraphX (Figure 5A) [43,44]. A 2D segmentation of the peel surface was extruded in 3D at a uniform thickness, with the cell edges rounded and cells connected like the idealized templates [12]. This simulation showed a stress at 20% strain of 2.88 MPa for the longitudinal stretch (Figure 5B dashed line, C) and 0.38 MPa for the transverse one (Figure 5B solid line, D). The tissue stiffness ratio of the template with realistic cell shapes is very similar (90.5%) to the one obtained from the stretching simulation of idealized cells with a 20-1 aspect ratio.

### 2.5. Estimating Cell-Wall Anisotropy

Although the simulations of turgid cells showed higher stiffness in the longitudinal direction, as in the experimental data, the stiffness ratio observed in silico in our realistic cell template was 4.43 times higher than the one observed in vivo. This suggests that the cell wall is softer in the longitudinal direction, even though it appears stiffer at the whole tissue scale. This supports the classical model of plant cell growth where the accumulation of cellulose microfibrils in the circumferential walls of the young rapidly elongating cells makes them stiffer in the transverse direction and able to expand less [45]. This demonstrates the importance of considering the effect of cell geometry and arrangements when interpreting whole-tissue experiments, as stiffness values and ratios do not directly map to cell wall stiffness and anisotropy.

To determine how much softer the walls need to be in the longitudinal direction to match the experiments, FEM modeling was performed on our realistic cell template with an anisotropic material model, a transversely isotropic model that allows the two orthogonal in-plane cell-wall directions to have different Young’s moduli. Compared to experimental data obtained from the osmotically treated plants (Figure 2B) to the stretching simulations of the onion template with isotropic material, the stress at 20% is very similar (92%) in the transverse direction, with 100 MPa providing a reasonable estimate for the Young’s modulus of the cell wall in this direction. This should not be confused with estimates of the Young’s modulus for entire tissues [42]. The Young’s modulus in the longitudinal direction was then fit to give a similar ratio (linearizing to account for Green strain) between the longitudinal and transverse directions. The simulation showed that lowering Young’s modulus in the longitudinal walls to 30% of the modulus in the transverse walls led to similar results to the microextensometer stretch in vivo. Thus, cellular geometry can affect the apparent tissue stiffness to such a degree that it makes the longitudinal direction appear stiffer, when in fact the cell wall is softer in that direction.

## 3. Materials and Methods

### 3.1. Plant Material and Sample Preparation

Spring onion (*Allium cepa* var. ‘White Lisbon’) seeds (Mr Fothergill’s 10890) were placed on Petri plates (Sigma-Aldrich, St. Louis, MO, USA, Z692344) containing wet filter paper and stratified at 4 °C for 3 days in darkness. Next, plates were kept in the growth chamber under long-day conditions (22 °C, 16 h light per day) for 7 days to allow the seeds to germinate, and seedlings were moved to pots (9 × 9 × 9 cm) and regularly watered. The epidermal peels were obtained from the bulbs (0.5 cm above the root) of mature onions (diameter > 1 cm) (Figure 1A). The outermost leaf was removed, and the second leaf was used with the epidermal peel taken from the adaxial side (Figure 1B,C). The dissected onion peels were kept in distilled water in a humid chamber prior to experimental work.

### 3.2. Microscopy and Image Processing

Onion sections were imaged using a Leica DM6000 microscope with objective ×1.25/0.04 for bright field mode and objective ×10/0.40 for DIC mode. The tailing mode was usedW to stitch different images and obtain overviews of entire sections. Cell and tissue section sizes were measured in Fiji [46].

To generate the epidermal templates, isolated epidermal sections were stained with propidium iodide 0.1% (Sigma-Aldrich, P4170) for 5 min and washed immediately with distilled water. Next, samples were imaged using a Leica TCS SP5 upright laser scanning confocal microscope with a water-dipper objective (×25/0.95). Excitation and emission wavelengths used were 535 nm and 617 nm, respectively. Confocal stacks were acquired at 1024 × 1024 resolution, with 0.5 μm distance in the Z-dimension. The fluorescence signal was projected into a mesh and cells were segmented with the image-processing software MorphoGraphX [43,44].

### 3.3. Microextensometer Setup

The microextensometer consists of a force sensor that records the force while pulling against the immobilized side of the sample with a robotic device (Figure S1). The force is measured with a 10 g force sensor (Futek, LSB200, Miniature S-Beam Jr. Load Cell, https://www.futek.com (accessed on 17 May 2022)) connected to the computer via a USB Digital Interface (19 bits resolution). The sensor is attached to a microrobotic actuator SmarAct (SmarAct GmbH, SLC-1780, http://www.smaract.com (accessed on 17 May 2022)) moving the sensor in 3 axes with linear piezo actuators (x-y-z). These actuators can record very small displacements down to several nanometers with closed-loop operation, but can also move up to several centimeters using a stick-slip mode when large displacements are required. The actuator robot can be manually operated by a control unit (SmarAct GmbH, MCS-, http://www.smaract.com (accessed on 17 May 2022)), which displays the current position of the sensor, and it can be controlled by a computer connected via USB.

For the experiments aiming to study high forces and performing tearing experiments (Figure S1), the microextensometer measurements were recorded with a digital microscope camera (DigiMicro 2.0, dnt GmbH, http://www.dnt.de (accessed on 17 May 2022)) connected to the computer via USB and controlled with the Cheese software (https://wiki.gnome.org/Apps/Cheese (accessed on 17 May 2022)), which allows the acquisition of images of the sample over the applied force. An additional light source was provided with LED rings (AmScope, http://www.amscope.com (accessed on 17 May 2022)) placed in the camera field view.

The microextensometer is operated by the fully automated MorphoRobotX software (https://www.MorphoRobotX.org (accessed on 17 May 2022)), which controls the positioner, records the change in force during displacement, and allows the extraction of sample stiffness.

### 3.4. Stretching the Samples with the Extensometer

The equipment and lights were switched on several hours before the experiment to stabilize thermal conditions. The onion epidermal sections were isolated and attached to pinched laboratory tags (Tough-Tags™, Diversified Biotech, Dedham, MA, USA) (Figure 1B). The average onion epidermal-section size was approximately 4 mm wide and 3.6 mm long. Next, samples were left in either water or 2.5 M NaCl baths in a humid chamber to stabilize the osmotic conditions of the sample (30 min immersion), as it could influence turgor pressure and cause cell deformation. The samples were attached to the microextensometer arms (Figure S1) and the extensometer process in MorphoRobotX was used to move the sensor against the immobilized arm and the force–displacement curves were acquired. The samples were kept hydrated by spraying the immersion solution on the surface prior stretching. For the microextensometer experiments of longitudinal and transverse displacements, comparisons were always performed within the same onion, the same leaf number, on the adaxial side and with sections of similar size. When using different samples, the same leaf number and onion stage of development (planted at the same time) was compared. The sample stiffness is measured as force per width over percentage of displacement.

### 3.5. Mechanical Modeling

#### 3.5.1. Idealized Cell Templates

Cellular templates of a tissue monolayer with idealized cell shapes (rounded cuboids) were generated for FEM modeling (Figure S5). Staggered or stacked 2D cell grids were generated with the CellMaker tool in MorphoDynamX (www.MorphoDynamX.org (accessed on 17 May 2022)). The cells were set to be 20 μm wide with aspect ratios of 1:1, 2:1, 5:1, 10:1 and 20:1, and vertices were generated with an average spacing of 2 μm. The size of the templates used in the simulation was 300 × 300 μm for the 1:1, 2:1, 5:1, 10:1; 600 × 600 μm for the 20:1. To create 3D cells, five segments, each 4 μm long, were extruded perpendicularly to the cell surface to create a 3D tissue. The anticlinal walls were set to be 20 μm long, and the walls triangulated. To recreate the curvature and shape of the real tissue, all but the central cell segments were smoothed. A pressure of 0.5 MPa was used for turgid samples and 0.01 MPa for the plasmolyzed one; pressure was applied to the interior faces of the cells. The templates were stretched to give a 50% increase in length.

#### 3.5.2. Generation of Realistic Cellular Templates

A cellular template for FEM modeling with realistic cell shapes of uniform thickness was generated from confocal images of onion epidermis. Following Sapala et al. [12], cells were segmented with MorphoGraphX, the CellMaker tool within MorphoDynamX was used to create extruded templates as was performed for the idealized cells. The size of the template obtained was 900 × 900 μm (Figure S6).

#### 3.5.3. Simulations of Extensometer Experiments

The FEM extensometer simulation was performed by modeling the cell wall as a hyperelastic material (Saint Venant–Kirchhoff material law), using triangular 3 node elements [9,11,17]. The equilibrium configuration was obtained with a pseudo-time-stepping approach that minimized the total potential energy with respect to the deformation $u=x-x_{0}$, independent of rigid body transformations. The energy is the sum of the strain-energy functional and the action of external forces—in this case turgor pressure—and is expressed as

$$\Pi \left(u\right)=W\left(E\left(u\right)\right)-P\int_{V_{0}}J\left(u\right)dv_{0}$$

where $W\left(E\left(u\right)\right)$ is the strain-energy functional, P is the pressure intensity, $V_{0}$ is the volume enclosed by the cells in the mesh subject to turgor pressure and $J\left(u\right)$ is the determinant of the deformation gradient. The form of the strain energy for a Saint Venant–Kirchhoff is

$$W\left(E\left(u\right)\right)=\int_{\Omega_{0}}\frac{\lambda }{2}\mathrm{Tr}{\left[E\left(u\left(x_{0}\right)\right)\right]}^{2}+\mu \mathrm{Tr}\left[E{\left(u\left(x_{0}\right)\right)}^{2}\right]d^{3}x_{0}$$

where $E\left(u\left(x_{0}\right)\right)$ is the strain tensor, which depends on the displacement vector $u=x-x_{0}$, and is expressed as a function of the coordinates of the undeformed body; and ${Ω}_{0}$ is the undeformed body domain. The two coefficients λ and µ are the Lamé coefficients, so related to Young’s modulus (E) and Poisson ratio (ν):

$$E=\frac{\mu \left(3\lambda +2\mu \right)}{\lambda +\mu },\;\nu =\frac{\lambda }{2\left(\lambda +\mu \right)}$$

For the computation of the equilibrium configuration by the FEM see [47], a pseudo-time-stepping implicit backward Euler scheme was adopted [11], with one increment of the solution computed as follows:

$${\hat{u}}_{\xi ,i}\left(t_{k+1}\right)={\hat{u}}_{\xi ,i}\left(t_{k}\right)-dt_{k}{\left(1+dt_{k}ℍ{\left(t_{k}\right)}_{\xi ,i;\theta ,j}\right)}^{-1}\frac{\partial \Pi }{\partial {\hat{u}}_{\theta ,\;j}}{│}_{t_{k}}$$

with Einstein summation convention, and where ${\hat{u}}_{\xi ,\;i}$ indicates the discretized nodal displacement at the node ξ in the space coordinate i, $t_{k}$ indicates the discretized time over which the solution updates are computed, 1 is the identity matrix, ${ℍ}_{\xi ,i;\theta ,j}$ indicates the Hessian of the total potential energy Π as a function of the discretized displacements, computed as

$${ℍ}_{\xi ,i;\theta ,j}=\frac{\partial^{2}\Pi }{\partial {\hat{u}}_{\xi ,\;i}\;\partial {\hat{u}}_{\theta ,\;j}}$$

The generalized forces are the first derivatives of the total potential energy with respect to discretized nodal displacements, and were computed analytically [48], while their second derivative (required for the Hessian) was computed numerically.

For the isotropic case, we used engineering strain (Biot strain) rather than more commonly used Green–Lagrange strain, as the first gives an approximately linear stress–strain curve when stretching an isotropic sheet of material and avoids the nonlinearities introduced by the quadratic form of the Green–Lagrange strain over large deformations. The Biot strain can be expressed as follows:

$$E_{Biot}=U-1$$

where U stands for the stretch tensor and 1 is the identity matrix. In the case of transversely isotropic material law, we used a transversely isotropic Saint Venant–Kirchhoff strain-energy function [49] with the traditional Green–Lagrange strain [17,36].

The extensometer simulations were performed by assigning Dirichlet boundary conditions to the nodes on the ends of the template in the axis of displacement. After each displacement step, the equilibrium was computed and the reaction force along the displacement direction was summed over the displaced nodes. The incremental displacement and reaction forces were logged into a file.

#### 3.5.4. Model Parameters

In the literature, experimental measurements of the Young’s modulus are available only for the outer periclinal walls of the abaxial epidermis (3.7 GPa longitudinal and 4.9 transverse directions) [40] and for the whole adaxial epidermis (59 MPa) [39]. Thus, intermediate values of the modulus were used for the simulation in accordance with Natonik-Bialon et al. [36]. The Young’s modulus was assigned as 100 MPa both in the longitudinal and transverse directions and the direction normal to the wall for the isotropic simulation. A Poisson’s ratio of 0 was used to eliminate any nonlinear effects from the geometry. The anisotropic simulation was set with a Young’s modulus of 30 MPa for the longitudinal direction and 100 MPa for the transverse and normal directions. A uniform pressure of 0.5 MPa for turgid samples, resulting in a cell volume increase of approximately 12%. FEM simulations of stretching were performed using the MorphoMechanX (www.MorphoMechanX.org (accessed on 17 May 2022)) simulation software [36]. The templates were stretched to 20% their original size.

## 4. Conclusions

We performed extensometer experiments on epidermal sections from growing spring onions. In agreement with previous results, the peels were stiffer when cells were oriented in the longitudinal direction, compared to sections with cells oriented in the transverse direction. This is in contrast with models of growth that predict the tissue to be softer in the primary direction of growth in elongating cells. Mechanical simulations of our extensometer experiments using pressurized 3D cellular templates showed that with uniform turgor pressure and uniform, isotropic material properties, the tissue appears much stiffer in the longitudinal direction if the cells are elongated. In the extensometer experiments, the cells were approximately 1.69 times stiffer in the longitudinal direction, whereas in the simulation with the onion template, the cells were 7.58 times stiffer. This suggests that geometry can have a greater effect than the stiffness difference, and that in fact the cell wall must be anisotropic, and softer in the longitudinal direction. We were able to approximate this difference by comparing the simulations to the forces observed experimentally and found a fit when the cell wall was 3.3 times softer in the longitudinal versus the transverse directions. In addition to a difference in stiffness, the strain–stress curves in the simulations had a large difference in shape compared to experimental curves. The stretching in the longitudinal direction in vivo was almost linear, with a slight reduction in stiffness for large strains, likely indicating plastic (irreversible) deformation at large strains. In the longitudinal direction, the simulations were almost linear as well. In the transverse direction, the simulations showed a strain-stiffening behavior, even when the cell wall was modeled with an isotropic material. This is likely from the flattening of the cells as they are stretched, which has a much larger effect in the transverse direction since there are more cell–cell contacts. In our experiments, we saw a slight strain-stiffening behavior, which was not as strong as the simulation. Possible reasons for this could be that the thick outer wall has a greater influence on the measurements [16], masking the effect of the geometry of the softer cell walls underneath. It would be informative to perform similar tests on the isolated outer epidermal cell wall [50], to directly analyze the anisotropy and nonlinear behavior of the outer cell wall over large deformations. Another possible explanation for the difference is that strain-softening plastic behavior induced by large deformations is masking the strain-stiffening contribution from the geometry. In more mature onion cells (from bulbs), substantial strain stiffening has been reported in the transverse direction [42], which they do not see in the longitudinal direction. Our models suggest that this is likely due to the geometry of the cells rather than the material properties of the cell wall in the transverse direction.

Our simulations show that the shape and arrangement of cells can have as strong effect on the stiffness measured in extensometer experiments as the cell wall itself. In the case of onion epidermal peels, this explains why the primary growth direction appears stiffer in stretching experiments, even though the cell wall itself is likely softer in this direction.

## Data Availability

The data presented in this study are available at https://morphographx.org/data/ (accessed on 17 May 2022).

## Supplementary Materials

The following supporting information can be downloaded at: https://www.mdpi.com/article/10.3390/ijms23105651/s1.
