# Supplementary material for: How Cell Geometry and Cellular Patterning Influence Tissue Stiffness
Source: Int J Mol Sci. 2022 May 18;23(10):5651. doi: 10.3390/ijms23105651 (PMC9145195; doi:10.3390/ijms23105651)
Supplement: Supplementary file 1 [file ijms-23-05651-s001.zip › ijms-1573431-supplementary.pdf]

Supplementary Information

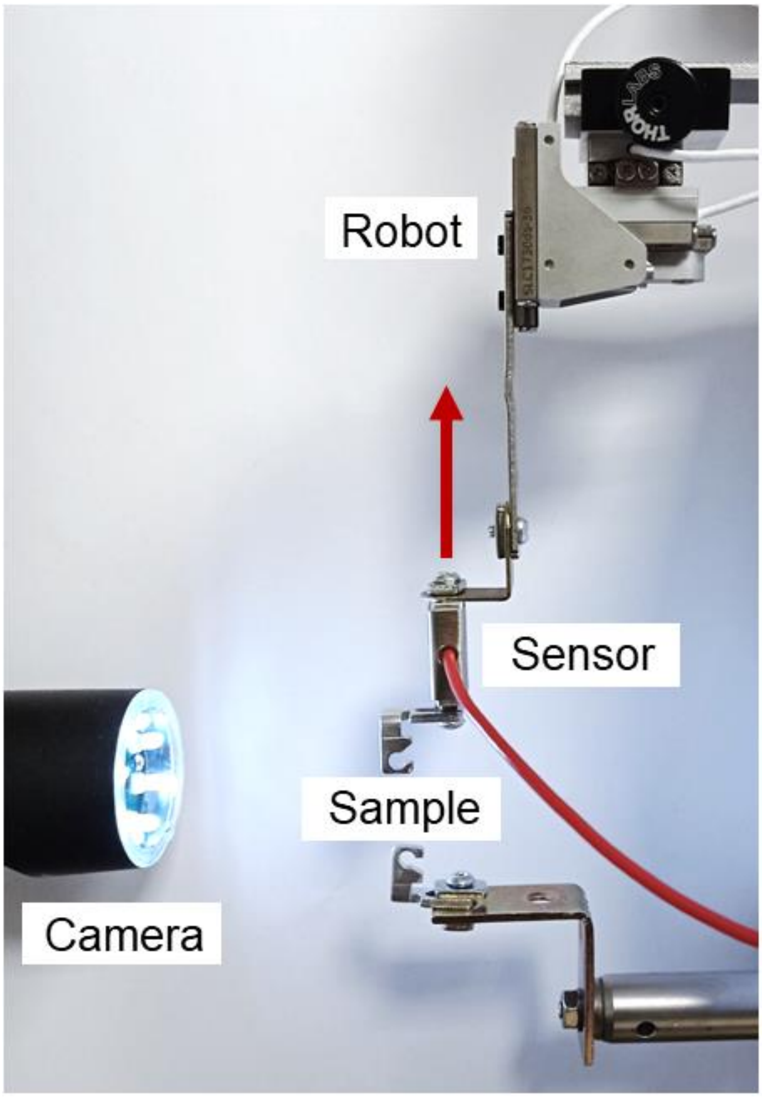

Figure S1. The micro-extensometer set up. The sample is mounted between a pair of hooks with the bottom hook connect to a fixed base and top hook connected to the force sensor. The robot moves against a fixed base to stretch the sample while the sensor measures the force. The experiment is monitored with a side camera.

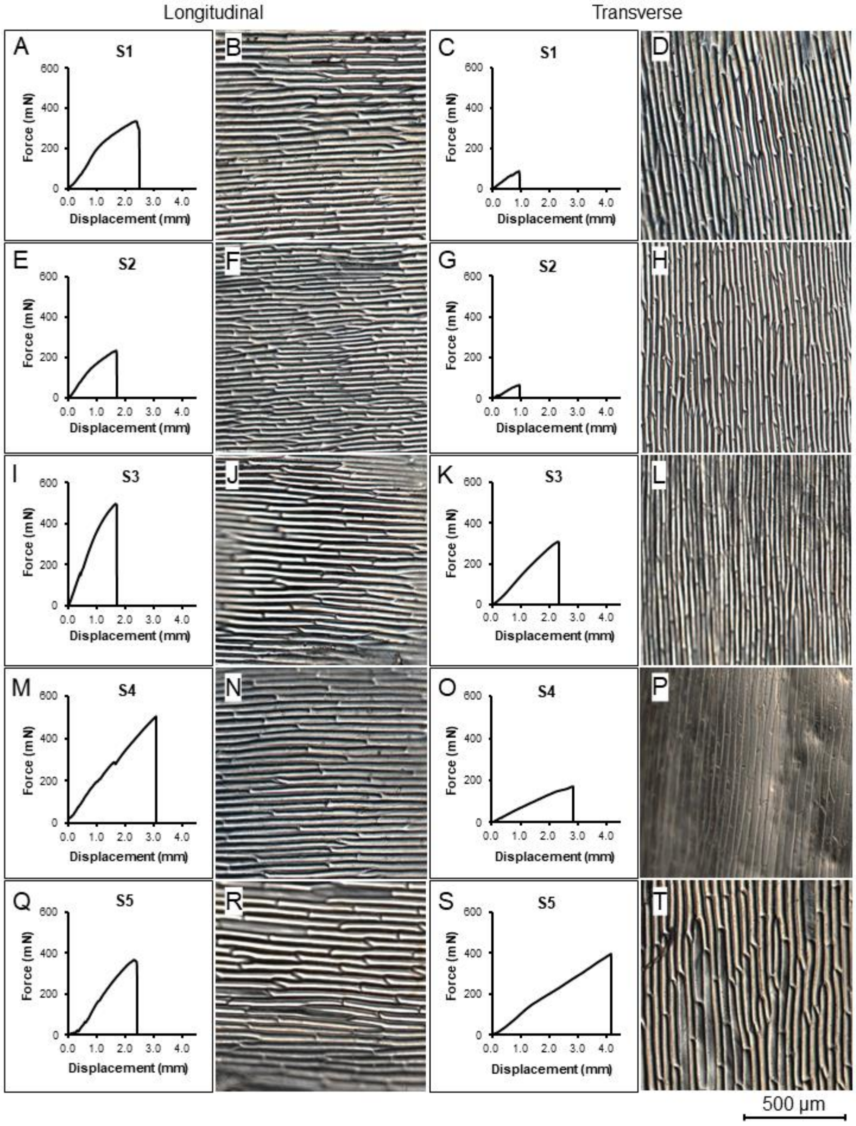

Figure S2. Raw force-displacement curves and cell sizes of turgid onion epidermal cells used for the stretching experiment. (**A**,**E**,**I**,**M**,**Q**) Force-displacement curves for samples stretched in the longitudinal direction and corresponding images (**B**,**F**,**J**,**N**,**R**) illustrating cell sizes in stretched samples. (**C**,**G**,**K**,**O**,**S**) Force-displacement curves for samples stretched in the transverse direction and corresponding images (**D,H,L,P,T**) illustrating cell sizes in stretched samples. Samples in rows were collected from the same onion and leaf number, with sections of similar size. All samples are taken from the adaxial side of onions at similar stage of development. The scale bar is shown in the figure. Force is displayed as mN and displacement as mm, S stands for sample number.

Table S1. Size of turgid onion epidermal sections. Length and width values represent the average between two sides.

Sample	Stretching orientation	x length (μm)	y width (μm)
1	Longitudinal	2899.85	3155.15
	Transverse	2875.9	2814.9
2	Longitudinal	2953.55	3317.4
	Transverse	3209.85	2835.35
3	Longitudinal	4998.5	5643.1
	Transverse	3717.2	5735.25
4	Longitudinal	2865.4	2806.05
	Transverse	2454.75	2636.4
5	Longitudinal	3784.15	5076.35
	Transverse	4913.05	5296.5

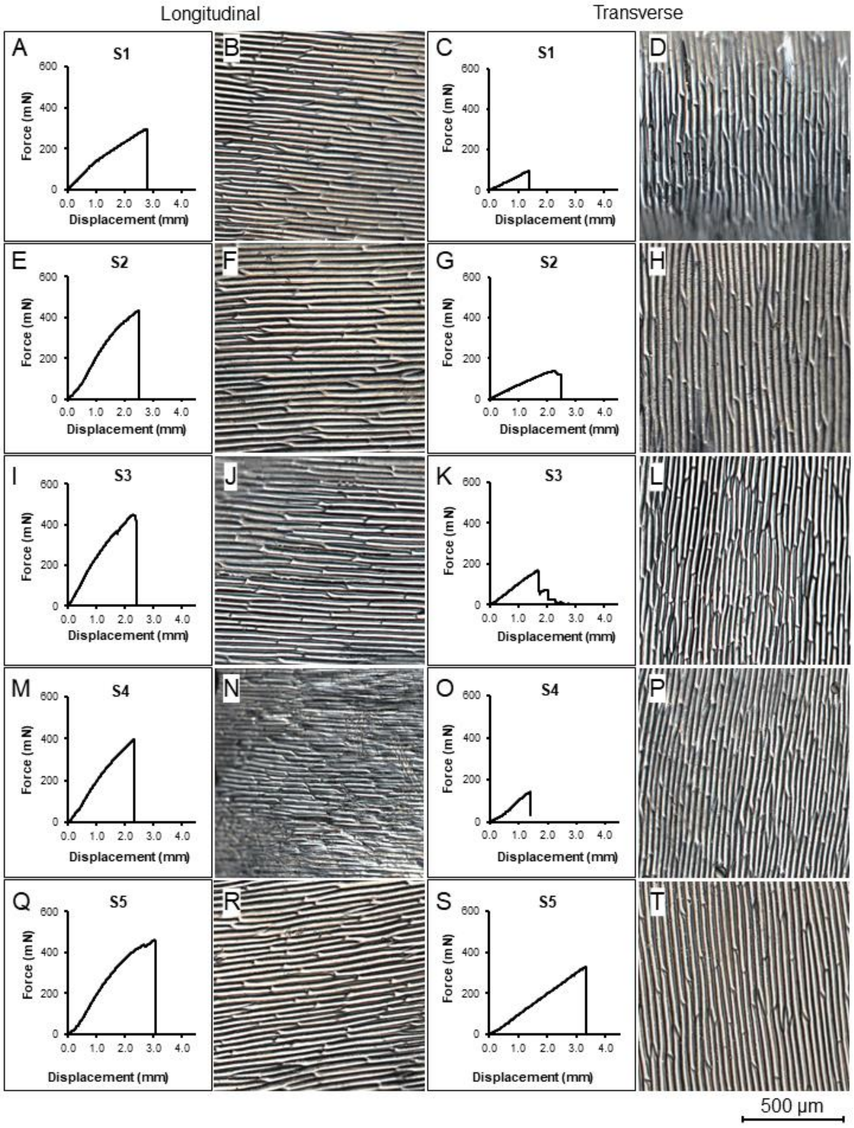

Figure S3. Raw force-displacement curves and cell sizes of osmotically treated onion epidermal cells used for the stretching experiment. (**A,E,I,M,Q**) Force-displacement curves for samples stretched in the longitudinal direction and corresponding images (**B,F,J,N,R**) illustrating cell sizes in stretched samples. (**C,G,K,O,S**) Force-displacement curves for samples stretched in the transverse direction and corresponding images (**D,H,L,P,T**) illustrating cell sizes in stretched samples. Samples in rows were collected from the same onion and leaf number, with sections of similar size. All samples are taken from the adaxial side of onions at similar stage of development. The scale bar is shown in the figure. Force is displayed as mN and displacement as mm, S stands for sample number.

Table S2. Size of osmotically treated onion epidermal sections. Length and width values represent the average between two sides.

Sample	Stretching orientation	x length (μm)	y width (μm)
1	Longitudinal	2777.6	2239.95
	Transverse	2129.5	1903.1
2	Longitudinal	3607.45	3561.95
	Transverse	3811.2	3642.05
3	Longitudinal	3576.75	5019.4
	Transverse	4760.25	5069.9
4	Longitudinal	3561.1	4291.45
	Transverse	3688.35	3610.8
5	Longitudinal	4373.9	5693.45
	Transverse	5081.3	5603.45

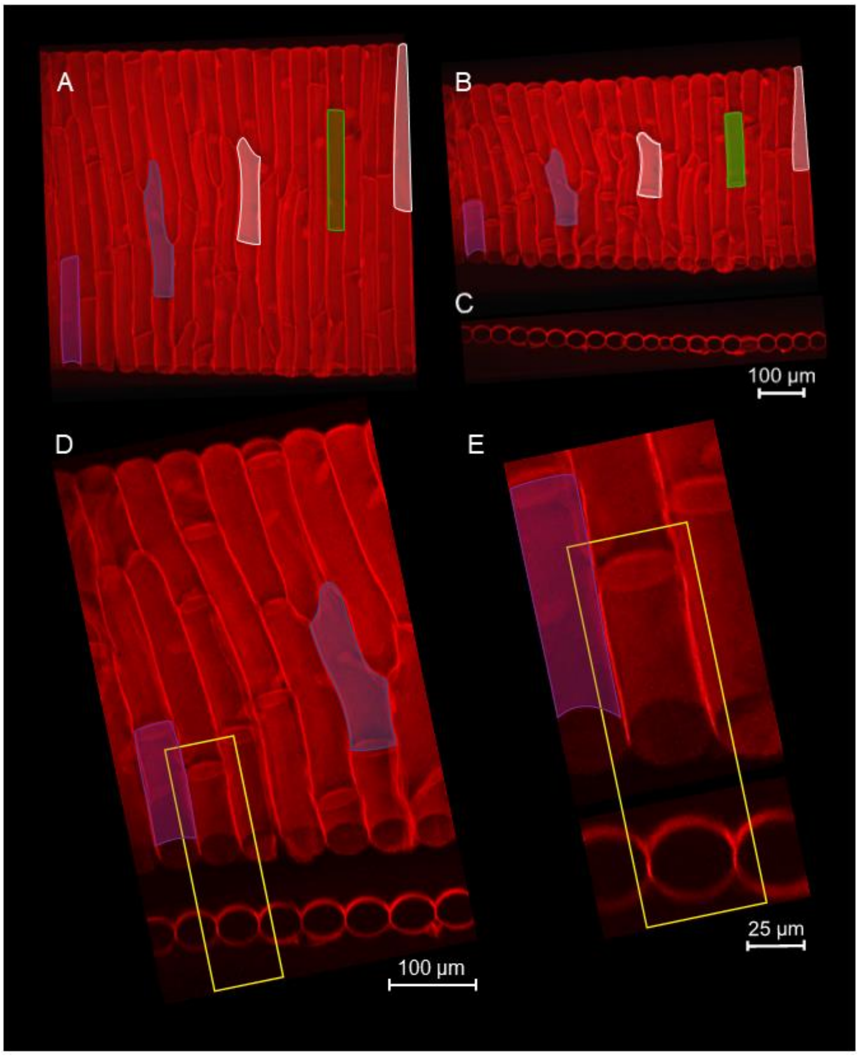

Figure S4. An adaxial epidermal peel stained with propidium iodide. (**A**) Top view. (**B**) Side view magnified in (**C,D,E**). Reference cells are highlighted in different colors or yellow frame. The scale bars are shown in the figure.

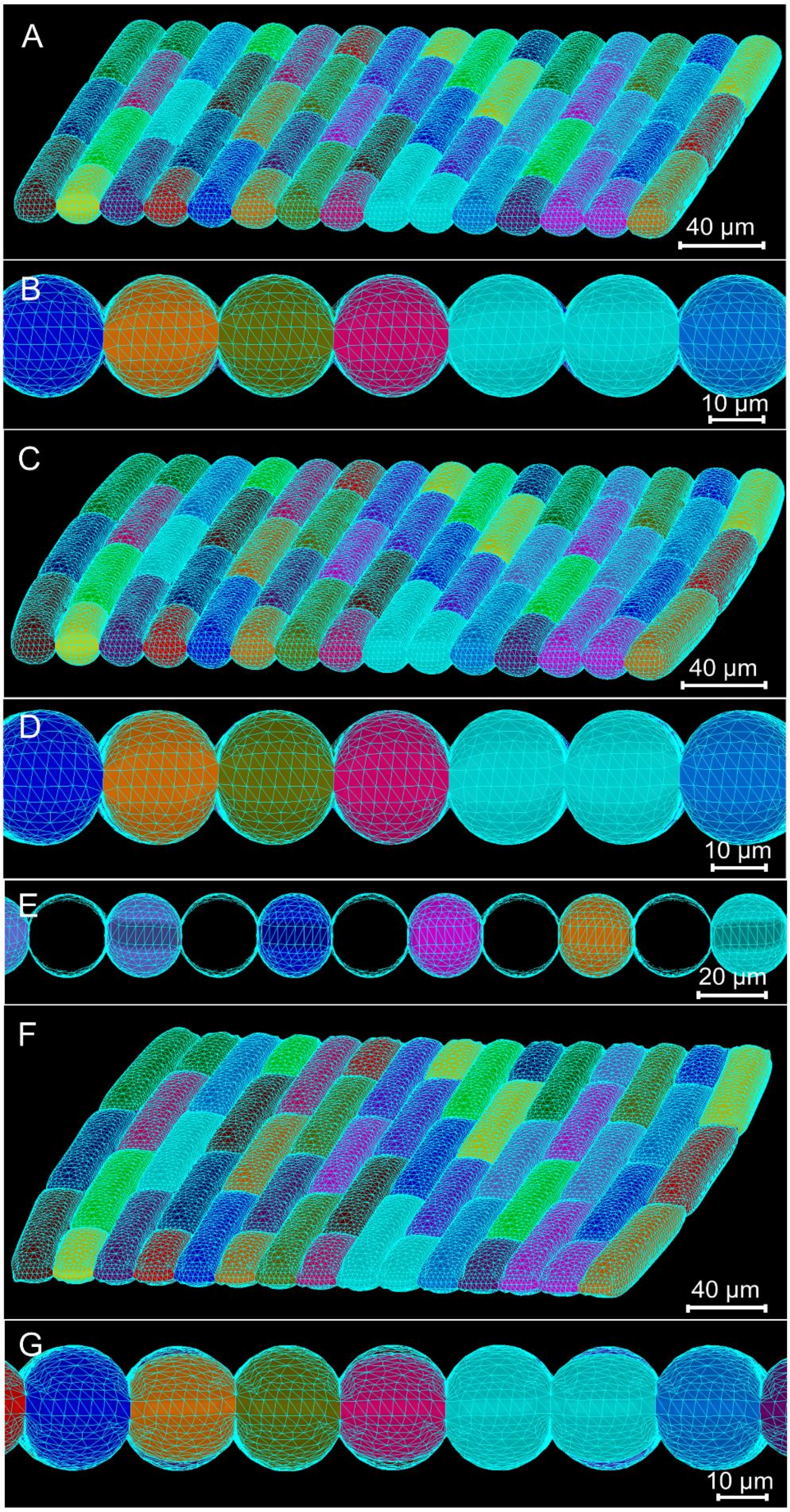

Figure S5. Idealized cell template with a 5-1 aspect ratio. (**A-B**) Non-pressurized tissue, (**C-E**) pressurized tissue, (**F-G**) stretched tissue, (**A,C,F**) surface view, (**B,D,G**) side view, (**E**) cross section. The scale bars are shown in the figure.

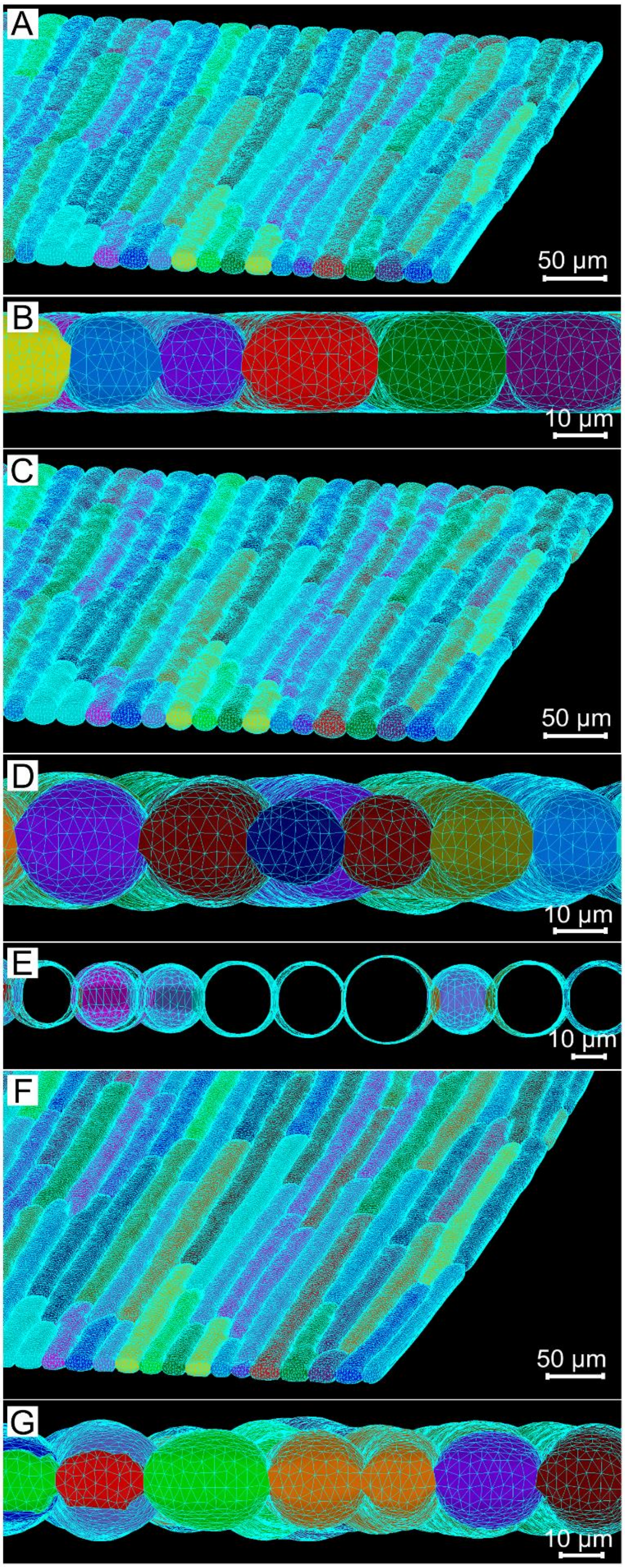

Figure S6. Realistic cell template. (**A-B**) Non-pressurized tissue, (**C-E**) pressurized tissue, (**F-G**) stretched tissue, (**A,C,F**) surface view, (**B,D,G**) side view, (**E**) cross section. The scale bars are shown in the figure.
